# Does advanced maternal age confer a survival advantage to infants born at early gestation?

**DOI:** 10.1186/1471-2393-13-87

**Published:** 2013-04-08

**Authors:** Sarka Lisonkova, Emmanuelle Paré, KS Joseph

**Affiliations:** 1 Department of Obstetrics & Gynaecology, University of British Columbia and the Children’s and Women’s Hospital of British Columbia, Vancouver, Canada; 2 School of Population and Public Health, University of British Columbia, Vancouver, Canada

**Keywords:** Advanced maternal age, Birth outcomes, Perinatal mortality, Fetuses-at-risk

## Abstract

**Background:**

Recent studies have shown that older mothers who deliver at preterm gestation have lower neonatal mortality rates compared with younger mothers who deliver at preterm gestation. We examined the effect of maternal age on gestational age-specific perinatal mortality.

**Methods:**

We compared fetal, neonatal and perinatal mortality rates among singleton births in the United States, 2003–2005, to mothers aged ≥35 versus 20–29 years. The analysis was stratified by gestational age and perinatal mortality rates were contrasted by maternal age at earlier (22–33 weeks) and later gestation (≥34 weeks). Gestational age-specific perinatal mortality rates were calculated using the traditional perinatal formulation (deaths among births at any gestation divided by total births at that gestation) and also the fetuses-at-risk model (deaths among births at any gestation divided by fetuses-at-risk of death at that gestation).

Logistic regression was used to estimate adjusted odds ratios (AOR) for perinatal death.

**Results:**

Under the traditional approach, fetal death rates at 22–33 weeks were non-significantly lower among older mothers (AOR 0.97, 95% confidence interval [CI] 0.91-1.03), while rates were significantly higher among older mothers at ≥34 weeks (AOR 1.66, 95% CI 1.56-1.76). Neonatal death rates were significantly lower among older compared with younger mothers at 22–33 weeks (AOR=0.93, 95% CI 0.88-0.98) but higher at ≥34 weeks (AOR 1.26, 95% CI 1.21-1.31). Under the fetuses-at-risk model, both rates were higher among older vs younger mothers at early gestation (AOR for fetal and neonatal mortality 1.35, 95% CI 1.27-1.43 and 1.31, 95% CI 1.24-1.38, respectively) and late gestation (AOR for fetal and neonatal mortality 1.66, 95% CI 1.56-1.76) and 1.21, 95% CI 1.14-1.29, respectively).

**Conclusions:**

Although the traditional prognostic perspective on the risk of perinatal death among older versus younger mothers varies by gestational age at birth, the causal fetuses-at-risk model reveals a consistently elevated risk of perinatal death at all gestational ages among older mothers.

## Background

The trend towards delayed childbearing has accelerated in industrialized countries during recent decades. In Canada, the mean maternal age at childbirth increased substantially in four decades from 23.7 years in 1969 to 29.4 years in 2009 [1,2]. More recently, the proportion of live births to women age 35 years or older in Canada doubled from 9.2% in 1991 to 18.3% in 2009 [1,2]. Similar changes were observed in the United States, where the average maternal age increased from 25.0 to 27.5 years between 1980 and 2009 [3,4], and the proportion of live births to mothers 35 years old or older increased from 4.9% in 1980 to 14.2% in 2009 [3,4]. Childbearing in industrialized countries in Europe, Australia and New Zealand followed the same trend [5-7].

While the trend towards delayed childbearing continues, the effect of maternal age on birth outcomes remains a subject of some controversy. Most studies have demonstrated an increased risk for preterm birth, intrauterine growth restriction, fetal death and neonatal death among singletons born to older mothers [8-10]. However, recent studies have suggested a favourable effect of advanced maternal age on neonatal death and serious neonatal morbidity among infants born at early gestation or low birth weight [11,12].

Our objective was to resolve the conflicting findings regarding the effect of advanced maternal age on fetal, neonatal and perinatal mortality at early gestation. We, therefore, carried out a study comparing gestational age-specific rates of fetal, neonatal and perinatal death among singleton infants born to mothers aged 20–29 years versus those born to mothers aged ≥35 years.

## Methods

We used population-based data on singleton births in the United States from the National Centre for Health Statistics (NCHS). Information in the NCHS birth/infant death and fetal death files was abstracted from birth certificates [13], with the birth-infant death linkage carried out by the NCHS (cohort linked birth-infant death file). The most recently available cohort files for the years 2003 to 2005 that linked infant deaths (up to 1 year of age) to birth certificates were used for the study. We included all infants born between 22 and 43 weeks of gestation, based on the clinical estimate of gestation at birth as this estimate is more accurate than gestational age based on menstrual dates [14-17]. This estimate of gestational age was provided by the health care provider, without specification of the source (i.e., whether based on clinical examination, ultrasound, etc.).

States that did not report the clinical estimate of gestational age were not included in the analysis. We also excluded births weighing less than 500 grams birth weight in order to avoid potential bias due to variable birth registration at the borderline of viability [18]. Information about maternal age and maternal and infant risk factors including education, race, parity, marital status, and infant’s gender was also obtained from NCHS files. Young mothers were defined as those aged 20–29 years at the time of birth, while older mothers were those aged 35 years or more. Fetal death was defined as death occurring before delivery, and neonatal death was defined as death during the first 28 days after birth. Perinatal death included both fetal and neonatal death (obstetric definition of perinatal death) [19].

Gestational age-specific fetal, neonatal, and perinatal rates were calculated using two different approaches: the traditional method and the fetuses-at-risk approach. Under the traditional method of calculating gestational age-specific fetal, neonatal and perinatal mortality, mortality rates were obtained by dividing the number of deaths at any gestation by the number of total births (or live births) at that gestation. Under the fetuses-at-risk approach, gestational age-specific mortality rates were calculated as the number of deaths at any gestation divided by the number of fetuses at risk of death at that gestation. Thus all fetuses in utero who were at risk of stillbirth were included in the denominator for gestational age-specific stillbirth rates [20,21]. Similarly, fetuses in utero who were at risk of birth and neonatal death at that gestation were included in the denominator for gestational age-specific neonatal death rates [21].

Logistic regression was used to estimate odds ratios and 95% confidence intervals and to adjust for confounders including maternal education (some college education vs high school or less), marital status (unmarried vs married), parity (nulliparous vs multiparous women, based on the total number of previous births), race (non-Hispanic white vs African American, Hispanic and other), smoking during pregnancy, infant’s gender and congenital anomalies (yes/no). To contrast the effects of maternal age on perinatal outcomes at early versus later gestation, two separate models were constructed for births at early (22–33 weeks) and late gestation (34–43 weeks). Under the traditional approach, logistic regression models for early gestation fetal, neonatal or perinatal death included only live births and fetal deaths at 22–33 weeks. In contrast, under the fetuses-at-risk approach, all ongoing pregnancies at 22 weeks gestation (i.e. all births at ≥22 weeks) were included in the logistic regression models examining death at early gestation. In this early gestation model, the outcome included fetal death, neonatal death or perinatal death at 22–33 weeks gestation only. Fetal and neonatal deaths and all live births that occurred after 33 weeks gestation were treated as survivors in this early gestation model and censored at 33 weeks. Logistic regression models examining fetal, neonatal and perinatal death at later gestation (≥34 weeks) included all ongoing pregnancies at 34 weeks gestation. The numerator and denominators were identical under the traditional and fetuses-at-risk approach in this analysis, as the total number of births at ≥34 weeks represented the number ongoing pregnancies at 34 weeks gestation. The only difference between the two approaches was for neonatal death; only live births were included in the denominator under the traditional model, whereas all births were included in the fetuses-at-risk formulation.

This study was exempted from ethics approval as analyses were performed on publicly accessible de-identified data. All analyses were carried out using SAS software, version 9.2 (SAS Institute Inc., Cary NC). A two-tailed P value <0.05 was considered significant.

## Results

There were 5,456,260 singleton births to mothers aged 20–29 years and 1,390,435 singleton births to mothers aged 35 years or more in the United States between 2003 and 2005. Fetal death rates were 3.1 per 1000 total births among younger mothers and 4.0 per 1000 total births among older mothers, while neonatal mortality rates were 2.5 per 1000 live births among younger mothers and 2.8 per 1000 live births among older mothers (Table 1). The rates of perinatal death were 5.6 among younger mothers and 6.8 per 1000 total births among older mothers. Older mothers were more likely to be married, multiparous, non-Hispanic white, non-smokers and educated as compared with younger mothers (Table 1). The gestational age distribution among older mothers was shifted towards lower gestational ages and older mothers were more likely to have had very low birth weight or high birth weight infants (P<0.001, Table 1).

**Table 1:** Maternal and fetal/infant characteristics by maternal age among singleton births, Unites States, 2003–2005

**Maternal age**	**20–29 years**		**≥35 years**		**P-value**
	**N**	**%**	**N**	**%**	
Total	5456260		1390435		
Primiparity^a^	1872836	34.5	220523	15.9	<0.001
Maternal race					<0.001
Non-Hispanic white	3148757	57.7	938771	67.5	
African American	894564	16.4	158429	11.4	
Hispanic	1122755	20.6	192012	13.8	
Other	59252	1.1	192012	0.6	
Missing race	230932	4.2	93055	6.7	
Unmarried	2233258	41.0	218518	15.7	<0.001
Education (any college)	2360637	43.7	966218	70.4	<0.001
Smoking during pregnancy	699909	12.8	92929	6.7	<0.001
Missing	231952	4.3	63329	4.6	
Gestational age (weeks)					<0.001
22–27	27605	0.5	7854	0.6	
28–33	90464	1.7	28120	2.0	
34–36	351685	6.4	98114	7.1	
37–40	4528206	83.0	1159833	83.4	
41–43	458300	8.4	96514	6.9	
Birth weight (g)					<0.001
500–999	28536	0.5	8528	0.6	
1000–2499	322682	5.9	85676	6.2	
2500–3499	3177955	58.2	720293	51.8	
3500–4499	1869180	34.3	550843	39.6	
≥4500	57907	1.1	25095	1.8	
Any congenital anomaly	63746	1.2	19276	1.4	<0.001
Infant's gender (male)	2793711	51.2	713245	51.3	0.046
Fetal death	16645	0.31	5583	0.40	<0.001
Neonatal death	13742	0.25	3900	0.28	<0.001
Perinatal death	30387	0.56	9483	0.68	<0.001

^a^ Based on total number of previous births.

Missing values of <1.5% were not listed in the table.

**Figure 1:**
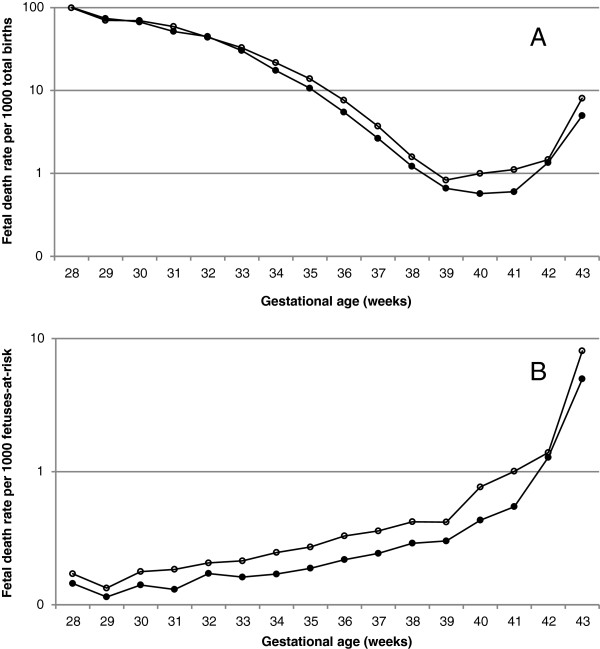
Gestational age-specific fetal death rates based on the traditional approach (A) and on the fetuses-at-risk approach (B) among women aged 20-29 years and ≥35 years, singleton births, United States, 2003-2005.

**Figure 2:**
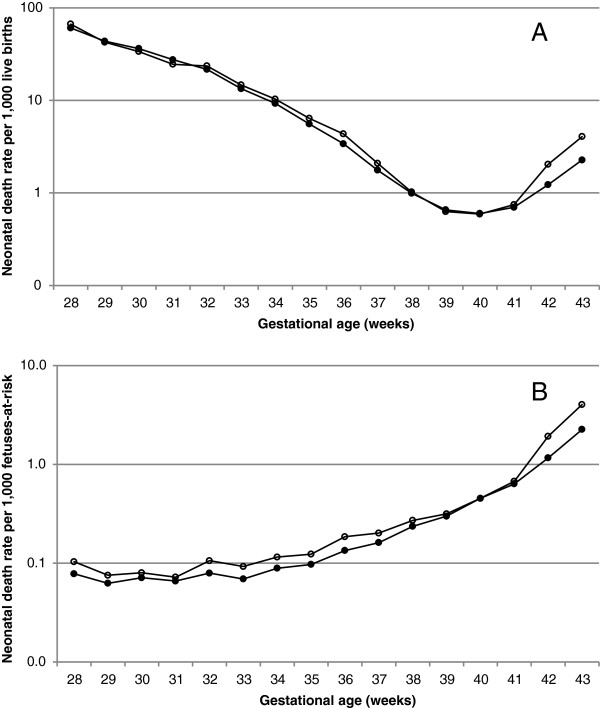
Gestational age-specific neonatal death rates based on the traditional approach (A) and on the fetuses-at-risk approach (B) among women aged 20-29 years and ≥35 years, singleton births, United States, 2003-2005.

**Figure 3:**
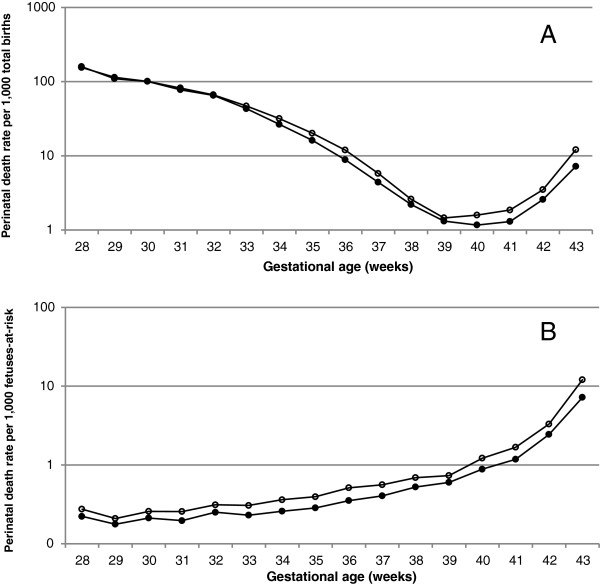
Gestational age-specific perinatal death rates based on the traditional approach (A) and on the fetuses-at-risk approach (B) among women aged 20-29 years and ≥35 years, singleton births, United States, 2003-2005.

Under the traditional approach, gestational age-specific fetal, neonatal and perinatal death rates declined with increasing gestation (Figures 1A, 2A, 3A, Table 2). The rate of fetal death at 22–33 weeks gestation was 76.4 per 1000 total births among mothers aged 35 years and older was and this was not significantly different from the fetal mortality rate at 22–33 weeks of 75.6 per 1000 total births among mothers aged 20–29 years (crude odds ratio 1.01, 95% CI 0.97-1.06, Table 3). However, fetal death rates at 34–43 weeks were substantially higher among older mothers compared with younger mothers (crude odds ratio 1.45, 95% CI 1.39-1.51; Figure 1A, Tables 2 and 3). Figure 2 shows gestational age-specific neonatal death rates among older vs younger mothers at early gestation (Table 2). The rate of neonatal death at 22–33 weeks gestation was 67.4 per 1,000 live births among mothers aged 35 years or older and this was significantly lower than the neonatal mortality rate of 72.9 per 1,000 live births among mothers 20–29 years of age (odds ratio 0.93, 95% CI 0.88-0.97, Table 3). On the other hand, neonatal mortality rates at later gestation (34–43 weeks) were higher among older mothers compared with younger mothers (odds ratio 1.13, 95% CI 1.07-1.20; Table 3). Adjustment for confounders strengthened the relationships between older maternal age and perinatal death but did not substantially change the results at early or late gestation (Table 3).

**Table 2:** Fetal and neonatal death rates per 1000 total/live births and per 1000 fetuses-at-risk (FAR) by maternal age, singleton births, United States, 2003–2005

**Gestational age (weeks)**	**Fetal deaths**		**Neonatal deaths**		**Total births**		**Fetuses-at-risk (FAR)**		**Fetal death rate per 1000 total births**		**Fetal death rate per 1000 FAR**		**Neonatal death rate per 1000 live births**		**Neonatal death rate per 1000 FAR**	
	**20-29 years**	**≥35 years**	**20-29 years**	**≥35 years**	**20-29 years**	**≥35 years**	**20-29 years**	**≥35 years**	**20-29 years**	**≥35 years**	**20-29 years**	**≥35 years**	**20-29 years**	**≥35 years**	**20-29 years**	**≥35 years**
22	866	249	986	234	2112	566	5456260	1390435	410.0	439.9	0.16	0.18	791.3	738.2	0.18	0.17
23	852	298	1458	395	3367	985	5454148	1389869	253.0	302.5	0.16	0.21	579.7	575.0	0.27	0.28
24	713	186	1300	349	4809	1273	5450781	1388884	148.3	146.1	0.13	0.13	317.4	321.1	0.24	0.25
25	569	160	865	214	5167	1473	5445972	1387611	110.1	108.6	0.10	0.12	188.1	163.0	0.16	0.15
26	636	175	567	171	5856	1689	5440805	1386138	108.6	103.6	0.12	0.13	108.6	112.9	0.10	0.12
27	632	191	480	150	6294	1868	5434949	1384449	100.4	102.2	0.12	0.14	84.8	89.4	0.09	0.11
28	784	236	424	143	7785	2378	5428655	1382581	100.7	99.2	0.14	0.17	60.6	66.8	0.08	0.10
29	620	184	339	104	8396	2627	5420870	1380203	73.8	70.0	0.11	0.13	43.6	42.6	0.06	0.08
30	761	244	385	110	11375	3509	5412474	1377576	66.9	69.5	0.14	0.18	36.3	33.7	0.07	0.08
31	703	253	356	99	13643	4289	5401099	1374067	51.5	59.0	0.13	0.18	27.5	24.5	0.07	0.07
32	924	282	427	145	20676	6437	5387456	1369778	44.7	43.8	0.17	0.21	21.6	23.6	0.08	0.11
33	865	291	371	126	28589	8880	5366780	1363341	30.3	32.8	0.16	0.21	13.4	14.7	0.07	0.09
34	906	334	474	156	51984	15437	5338191	1354461	17.4	21.6	0.17	0.25	9.3	10.3	0.09	0.12
35	993	363	513	165	93283	26198	5286207	1339024	10.6	13.9	0.19	0.27	5.6	6.4	0.10	0.12
36	1132	431	697	243	206418	56479	5192924	1312826	5.5	7.6	0.22	0.33	3.4	4.3	0.13	0.19
37	1212	450	805	253	457804	121419	4986506	1256347	2.6	3.7	0.24	0.36	1.8	2.1	0.16	0.20
38	1310	476	1067	307	1075784	300718	4528702	1134928	1.2	1.6	0.29	0.42	1.0	1.0	0.24	0.27
39	1040	348	1031	263	1571669	418969	3452918	834210	0.7	0.8	0.30	0.42	0.7	0.6	0.30	0.32
40	812	318	852	188	1422949	318727	1881249	415241	0.6	1.0	0.43	0.77	0.6	0.6	0.45	0.45
41	250	97	291	65	416096	87152	458300	96514	0.6	1.1	0.55	1.01	0.7	0.7	0.63	0.67
42	54	13	49	18	39988	8866	42204	9362	1.4	1.5	1.28	1.39	1.2	2.0	1.16	1.92
43	11	4	5	2	2216	496	2216	496	5.0	8.1	4.96	8.06	2.3	4.1	2.26	4.03
Total	16645	5583	13742	3900	5456260	1390435	5456260	1390435	3.1	4.0	3.05	4.02	2.5	2.8	2.52	2.80

**Table 3:** Gestational age-specific fetal, neonatal and perinatal death rates and odds ratios comparing mothers ≥35 years vs 20–29 years, singleton births, United States, 2003–2005

	**Traditional model**				**Fetuses-at-risk model**			
	**Rate per 1000 total births**^**a**^		**Unadjusted odds ratio (95% CI)**	**Adjusted odds ratio (95% CI)**	**Rate per 1000 fetuses-at-risk**		**Unadjusted odds ratio (95% CI)**	**Adjusted odds ratio (95% CI)**
	**age 20–29 years**	**age ≥35 years**			**age 20–29 years**	**age ≥35 years**		
**Fetal death**								
22-33 weeks	75.6	76.4	1.01 (0.97- 1.06)	0.97 (0.91-1.03)	1.6	2.0	1.21 (1.16- 1.26)	1.35 (1.27-1.43)
34-43 weeks	1.4	2.1	1.45 (1.39- 1.51)	1.66 (1.56-1.76)	1.4	2.1	1.45 (1.39- 1.51)	1.66 (1.56-1.76)
Total	3.1	4.0	1.32 (1.28- 1.36)	1.48 (1.42-1.55)	3.1	4.0	1.32 (1.28- 1.36)	1.48 (1.42-1.55)
**Neonatal death**								
22-33 weeks	72.9	67.4	0.93 (0.88- 0.97)	0.93 (0.88-0.98)	1.5	1.6	1.11 (1.05- 1.16)	1.31 (1.24-1.38)
34-43 weeks	1.1	1.2	1.13 (1.07- 1.20)	1.21 (1.14-1.29)	1.1	1.2	1.13 (1.07- 1.19)	1.21 (1.14-1.29)
Total	2.5	2.8	1.12 (1.08- 1.16)	1.26 (1.21-1.31)	2.5	2.8	1.11 (1.08- 1.15)	1.26 (1.21-1.31)
**Perinatal death**								
22-33 weeks	143.0	138.7	0.97 (0.93- 1.00)	0.94 (0.90-0.98)	3.1	3.6	1.16 (1.12- 1.20)	1.33 (1.27-1.38)
34-43 weeks	2.5	3.3	1.31 (1.27- 1.36)	1.42 (1.37-1.49)	2.5	3.3	1.31 (1.27- 1.36)	1.42 (1.37-1.49)
Total	5.6	6.8	1.23 (1.20- 1.25)	1.37 (1.33-1.41)	5.6	6.8	1.23 (1.20- 1.25)	1.37 (1.33-1.41)

^a^ Neonatal death rates expressed per 1000 live births.

Under the fetuses-at-risk approach, gestational age-specific fetal, neonatal and perinatal death rates increased with increasing gestational age (Figures 1B, 2B, 3B). Fetal death rates were higher among older compared with younger mothers (Figures 1B, 2B and 3B, Table 2, Table 3) at both early gestation (odds ratio 1.21, 95% CI 1.16-1.26) and at later gestation (odds ratio 1.45, 95% CI 1.39-1.51). A similar association was observed for neonatal death rates, with older mothers having higher rates of neonatal death than younger women at early gestation (odds ratio 1.11, 95% CI 1.05-1.16) and at late gestation (odds ratio 1.13, 95% CI 1.07-1.19). Adjustment for potential confounders increased the strength of the association between older maternal age and fetal and neonatal mortality at early and late gestation (Table 3).

## Discussion

Our study showed that under the traditional perinatal model, fetal mortality rates at early gestation (22–33 weeks) were non-significantly lower among older vs younger mothers, while neonatal mortality and perinatal mortality rates at early gestation were significantly lower among older women. The opposite was true at later gestation (34–43 weeks), with fetal, neonatal and perinatal mortality rates being higher among older mothers compared with younger mothers. In contrast, the fetuses-at-risk model showed a more consistent picture with older mothers having higher rates of fetal, neonatal and perinatal death at all gestational ages.

Our results provide a comprehensive overview of the findings of previous studies including those restricted to births at early gestation [11], and low birth weight births [12]. Further, our study shows that maternal age contrasts under the tradition perinatal model result in intersecting mortality curves if perinatal death rates among older vs younger mothers are contrasted at early and late gestation. The phenomenon of intersecting perinatal mortality curves has been demonstrated for numerous other determinants including maternal smoking, parity, plurality, infant sex, altitude, race, pregnancy complications such as hypertension, etc [22-30]. Thus the apparent survival advantage that preterm infants of older mothers appear to enjoy is also seen among preterm/low birth weight infants of mothers who smoke compared with preterm/low birth weight infants of mothers do not smoke [29]. This same survival advantage is also observed among preterm/low birth weight infants of women with twin pregnancies (vs those who have singleton pregnancies) [24] and women who have pregnancy complications such as hypertensive disorders (versus those who do not have pregnancy complications) [30]. Although it is tempting to explain away the survival advantage in each of these contrasts with ad hoc explanations, such intersecting perinatal mortality curves are a general phenomenon and the ideal explanation should seek to parsimoniously address all these various contrasts. The fetuses-at-risk approach is a general solution to this paradox in so far as it provides a biologically plausible and consistent result; under this formulation smokers have higher perinatal mortality rates compared with non-smokers at all gestational ages [29], twins have higher perinatal mortality compared with singletons at all gestational ages [24] and women with hypertensive disorders have higher rates of perinatal mortality rates than women without hypertensive disorders at all gestational ages [30].

Disparate results obtained from traditional vs fetuses-at-risk approaches highlight two different perspectives: prognostic (i.e., predictive or acausal) and causal [21]. The traditional approach provides an accurate prognostic perspective for the newborn infant and has utility in predicting neonatal death. Under the traditional model, gestational age serves as an excellent predictor for neonatal mortality and infants born to older mothers at early gestation are observed to have a more favourable *prognosis* as compared with infants born to younger mothers. The fetuses-at-risk model, on the other hand, provides insights into the biologic role of maternal age (and other factors responsible for intersecting perinatal mortality curves such as smoking, plurality, hypertension in pregnancy) from a fetal perspective. It has little prognostic value but represents a causal model [31,32] which suggests that older maternal age is *causally* associated with a higher risk of fetal and neonatal death at early and late gestation. Our study also demonstrates that (causal) etiologic studies restricted to births at early gestation (or low birth weight births) may yield biased results if they use traditional measures of gestational age-specific perinatal mortality since they ignore the biologic continuum of pregnancy [11,12,33,34]. Whereas results based on the traditional model yield valid *prognostic* estimates, any *causal* inference based on these associations (whether related to the effect or older maternal age, smoking or hypertensive disorders) is likely biased. The reason for the acausal nature of traditional models and the causal nature of the fetuses-at-risk model relates to how these two models treat gestational age: in the traditional model gestation age is a determinant, whereas in the fetuses-at risk model it represents survival time [27,31]. Treating gestational age as survival time permits the estimation of incidence rates which are central to causal inference.

While the fetuses-at-risk-based measures of gestational age-specific perinatal mortality represent a causal model, there are some conceptual challenges regarding the optimal analytical approach to model these time-to-event perinatal data [35]. The Cox model constitutes a robust analytical tool for multivariable survival analysis which assumes that censoring is non-informative. This creates a significant problem in perinatal studies wherein stillbirth and live birth represent competing risks in studies of stillbirth (and stillbirth and neonatal death represent competing risks in studies of neonatal death). The competing risk issue between stillbirth and neonatal death is simply resolved by using perinatal death as an outcome, though there are socio-cultural reasons that may require studies to focus on stillbirths or neonatal deaths separately. Logistic regression analyses based on fetuses-at-risk approach and focusing on perinatal death (as done in this study) offer a practical analytical approach to avoiding some of these problems and also address the potential bias introduced by the traditional approach when examining causal associations at early gestation [36].

The strength of our study includes the use of a large population-based dataset with information collected according to uniform protocols across the United States [13]. The limitations include some degree of data inaccuracy that is inevitable in large administrative databases. The ascertainment of gestational age in vital statistics has been traditionally based on menstrual dating, although a clinical estimate of gestation based on early ultrasound is available for recent years [14]. In this study we used the more accurate clinical estimate of gestation and excluded births without such an estimate [14-17]. Some congenital anomalies were likely underreported and this may have affected some estimates in our study. We did not have information on socio-economic status, body mass index and behavioural factors such as substance use or alcohol consumption that are known to be associated with maternal age and perinatal outcomes. Finally, we ascribed time of fetal or neonatal death to the time of birth (in gestational weeks). The former assumption was made because time of fetal death was not available in our data source. For neonatal deaths, we assumed an antepartum cause of death among infants who died during the neonatal period. The time between birth and neonatal death was therefore considered a latent period between the birth of a compromised fetus and neonatal death.

## Conclusion

In summary, our study demonstrates that contrasts of traditional gestational age-specific perinatal mortality rates among older versus younger mothers result in the intersecting perinatal mortality curves. The traditional perinatal model provides a varying prognostic perspective on the effect of older maternal age, with protection against neonatal/perinatal death observed at early gestation and an increased risk of neonatal/perinatal death observed at later gestation. On the other hand, the fetuses-at-risk model provides a consistent causal perspective on the effect of older maternal age, with an increased risk of fetal, neonatal and perinatal death observed at all gestational ages. This underscores the need for a proper conceptualization of gestational age-specific perinatal mortality in studies examining causal associations.

## Abbreviations

AOR: Adjusted odds ratio; NCHS: National Centre for Health Statistics.

## Competing interests

None of the authors have a personal financial relationship relevant to this article.

## Authors’ contribution

SL and KSJ contributed to the conception and design of the study, SL conducted the data analysis and drafted the manuscript and EP and KSJ revised the manuscript for intellectual content; all authors approved the final version of the manuscript.

## Pre-publication history

The pre-publication history for this paper can be accessed here:

http://www.biomedcentral.com/1471-2393/13/87/prepub
